# The Pathways Underlying the Multiple Roles of p62 in Inflammation and Cancer

**DOI:** 10.3390/biomedicines9070707

**Published:** 2021-06-22

**Authors:** Paulina Hennig, Gabriele Fenini, Michela Di Filippo, Tugay Karakaya, Hans-Dietmar Beer

**Affiliations:** 1 Department of Dermatology, University Hospital Zurich, Wagistrasse 18, CH-8952 Schlieren, Switzerland; Paulina.Hennig@usz.ch (P.H.); Gabriele.Fenini@usz.ch (G.F.); Michela.Difilippo@usz.ch (M.D.F.); Tugay.Karakaya@usz.ch (T.K.); 2 Faculty of Medicine, University of Zurich, CH-8032 Zurich, Switzerland

**Keywords:** p62, autophagy, inflammasomes, Nrf2/Keap1, NF-κB, mTORC1, inflammation, cancer

## Abstract

p62 is a highly conserved, multi-domain, and multi-functional adaptor protein critically involved in several important cellular processes. Via its pronounced domain architecture, p62 binds to numerous interaction partners, thereby influencing key pathways that regulate tissue homeostasis, inflammation, and several common diseases including cancer. Via binding of ubiquitin chains, p62 acts in an anti-inflammatory manner as an adaptor for the auto-, xeno-, and mitophagy-dependent degradation of proteins, pathogens, and mitochondria. Furthermore, p62 is a negative regulator of inflammasome complexes. The transcription factor Nrf2 regulates expression of a bundle of ROS detoxifying genes. p62 activates Nrf2 by interaction with and autophagosomal degradation of the Nrf2 inhibitor Keap1. Moreover, p62 activates mTOR, the central kinase of the mTORC1 sensor complex that controls cell proliferation and differentiation. Through different mechanisms, p62 acts as a positive regulator of the transcription factor NF-κB, a central player in inflammation and cancer development. Therefore, p62 represents not only a cargo receptor for autophagy, but also a central signaling hub, linking several important pro- and anti-inflammatory pathways. This review aims to summarize knowledge about the molecular mechanisms underlying the roles of p62 in health and disease. In particular, different types of tumors are characterized by deregulated levels of p62. The elucidation of how p62 contributes to inflammation and cancer progression at the molecular level might promote the development of novel therapeutic strategies.

## 1. Introduction

**Figure 1:**
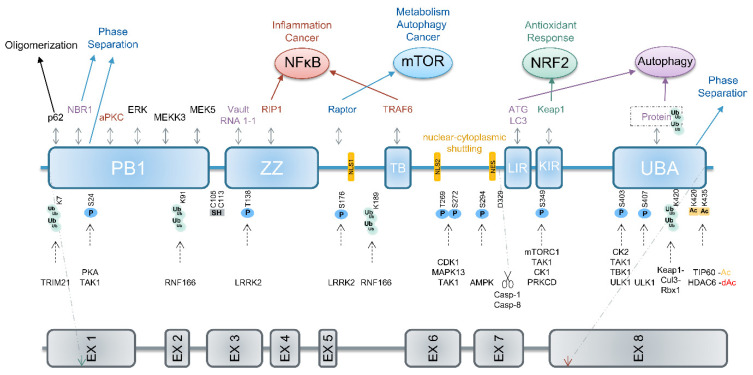
Structure of *SQSTM1* and p62. Domain architecture of p62, posttranslational modifications, binding/modifying proteins and pathways regulated by p62. The PB1 domain mediates oligomerization and activation of p62, UBA-mediated dimerization holds p62 inactive. For phase separation, the PB1 and UBA domains are required. The role of p62 as a cargo receptor in autophagy is mediated by the LIR motif, linking it to LC3 on autophagosomes, and the UBA domain, which binds to ubiquitinated cargos. Upon phosphorylation at Ser349, the KIR motif interacts with Keap1, thereby liberating Nrf2 and inducing Nrf2 target gene expression. When Raptor binds to p62, mTOR is activated inducing metabolic pathways. Binding of RIP1 to the ZZ or TRAF6 to the TB motif supports activation of NF-κB. Cleavage after Asp329 by caspase-8 (casp-8) or caspase-1 (casp-1) generates an amino terminal fragment of p62 activating mTOR. Disulfide bond formation involving Cys105 or Cys113 as well as binding of vault RNA1-1 via the ZZ motif supports autophagy. Please see in the text for details. Abbreviations: Ac: acetylation, dAc: de-acetylation, C: Cys, D: Asp, K: Lys, KIR: Keap1-interacting region, LIR: LC3-interacting region, NES: nuclear export signal/sequence, NLS: nuclear localization signal/sequence, PB1: Phox and Bem1p, S: Ser, T: Thr, TB: TRAF6-binding domain, UBA: ubiquitin-associated, ZZ: Zinc finger, [1,12,13,14].

*SQSTM1* (sequestosome 1), the gene coding for the p62 protein, spans about 16 kb and is located on chromosome 5 [1,2]. It is widely expressed and contains eight exons with a short 5′UTR (untranslated region) and an unusually long 3′UTR [3,4] (Figure 1). In humans, due to alternative splicing, *SQSTM1* gives rise to two different isoforms. Isoform 1 represents the full-length p62 protein with 440 amino acids, whereas isoform 2 lacks 84 amino acids at the amino terminus in the PB1 domain [5,6,7]. The expression of *SQSTM1* is stress-associated and induced by the transcription factors Nrf2, NF-κB, and MiT/TFE [8,9,10]. Apart from their roles in melanocytes, members of the MiT/TFE (microphthalmia/transcription factor E) family of helix-loop-helix transcription factors are activated by different types of internal and external stresses and also in cancer [11] (for Nrf2 see Section 4.2 and for NF-κB Section 4.4).

Deregulated *SQSTM1* expression is associated with several human diseases [12,15]. Missense mutations of *SQSTM1* cause ALS (amyotrophic lateral sclerosis), PDB (Paget’s disease of bone) or FTLD (frontotemporal lobar degeneration) [16]. ALS is a neurodegenerative disease of motor neurons. The role of p62 in ALS is most likely defined by its autophagy-dependent requirement for proteostasis [17], the dynamic regulation of a functional proteome [18]. Similarly, FTLD is caused by proteotoxic stress-induced neurotoxicity due to the absence of functional *SQSTM1* expression [19]. In PDB, a chronic progressive skeletal disorder, p62 mutations induce activation of osteoclasts in a non-physiological manner resulting in increased and deregulated bone turnover [20,21]. Other neurodegenerative diseases, such as Parkinson’s disease and Huntington’s disease, are also characterized by low levels of p62 [22]. In contrast, numerous types of cancer exhibit increased amounts of p62 in the tumor cells, including hepatocellular carcinoma (HCC, see Section 5, ovarian, breast, lung, or pancreatic cancer [1,5,23,24,25,26,27,28]. In these cells, high levels of p62 cause Nrf2 (Section 4.2), mTORC1 (Section 4.3), or/and NF-κB (Section 4.4) activation, which favors the development and proliferation of cancer cells. Interestingly, high levels of p62-positive inclusion bodies, termed Mallory–Denk bodies, are frequently found in patients with chronic liver conditions, such as HCCs, but also in hepatocellular neoplasms, alcoholic and non-alcoholic steatohepatitis, or metabolic disorders [29], and are associated with Nrf2 activation [23,30]. In contrast, p62 expression is reduced in the tumor stroma, for example in cancer-associated fibroblasts of prostate cancer cells [31] or in tumor-associated macrophages [32]. This causes metabolic reprogramming and secretion of IL-6 and TGF-β, both supporting growth and proliferation of tumor cells. Therefore, p62 expression might serve as a diagnostic and prognostic marker in carcinomas [12].

Ablation of expression in mice revealed physiological roles of p62 in osteoclastogenesis and bone remodeling, and in obesity and adipogenesis [33,34]. Upon aging, p62 knockout mice develop obesity caused by hyperphagia due to leptin resistance [35].

This review article aims to summarize what is known about the complex role of p62 in inflammation and inflammation-related diseases, including cancer. The focus is on the underlying molecular mechanisms and pathways. Section 2 describes the domain structure of p62, its post-translational modifications, and interacting proteins. In Section 3, we discuss the role of p62 as a receptor in autophagy and its regulation, and in Section 4, its involvement in signaling pathways, namely inflammasomes (Section 4.1), Nrf2 (Section 4.2), mTOR (Section 4.3), and NF-κB (Section 4.4). In Section 5, we address the mechanisms underlying deregulated expression in cancer and the consequences with HCCs as an example. Our article is based on a literature search using Pubmed (search criteria “p62” and “autophagy”, “inflammasome”, “Nrf2”, “mTOR”, “NF-kappaB” or “cancer/HCC”).

## 2. p62 Is a Posttranslationally Modified Multi-Domain Protein

p62 consists of 440 amino acids with several domains that influence different pathways upon interaction with distinct binding partners (Figure 1). The roles of p62 in five of these pathways are discussed in more detail below, including autophagy (Section 3), inflammasomes (Section 4.1), Keap1/Nrf2 (Section 4.2), mTORC1 (Section 4.3), and NF-κB (Section 4.4). The activity of p62 is regulated by posttranslational mechanisms, namely ubiquitination, phosphorylation, acetylation, proteolytic processing, and the formation of disulfide bridges (Figure 1).

The amino terminal PB1 domain (Phox1 and Bem1p) of p62 is required for the homotypic oligomerization of inactive p62 dimers, which results in a helical structure required for its role as a cargo receptor in autophagy [36]. The ubiquitin ligase TRIM21 (tripartite motif 21) inhibits oligomerization, and therefore activation of p62 upon ubiquitination via Lys63-linkage at Lys7 of p62. Consequently, ablation of TRIM21 results in Keap1 degradation, Nrf2 activation, and oxidative stress resistance (Section 4.2) [37].

Adjacent to the PB1 domain, p62 harbors a zinc finger (ZZ domain), frequently associated with DNA binding in other proteins. However, evidence for a direct binding of p62 to DNA is missing, although p62 can shuttle between nucleus and cytoplasm via two nuclear localization sequences (NLS1 and NLS2) and one nuclear export signal (NES) [38,39]. In contrast, p62 interacts with the small non-coding vault RNA1-1 via the ZZ domain favoring p62 oligomerization and autophagy [40]. RIP1 (receptor interacting protein 1, also receptor interacting serine/threonine kinase 1 or RIPK1) is an established binding partner of the ZZ domain and this interaction can induce NF-κB activation [41]. Moreover, p62 interacts via its TB domain (TRAF binding domain), located between NLS1 and NLS2, with the E3 ubiquitin ligase TRAF6 (tumor necrosis factor receptor associated factor 6), also leading to NF-κB activation [42].

LC3 (microtubule-associated protein 1A/1B-light chain 3) is a central autophagy protein and interacts with the LIR (LC3-interacting region) of p62, a motif commonly located in autophagy receptors [43,44]. As the carboxy terminal UBA (ubiquitin associated) domain binds and thereby recruits ubiquitinated proteins designated for autophagosomal degradation, p62 functions as a cargo receptor for selective autophagy.

Several kinases, such as mTORC1, TAK1 (TGF-β-activated kinase), CK1 (casein kinase 1), and PKC-delta (protein kinase C delta, PRKCD), phosphorylate p62 at Ser349 [45,46,47,48]. Then, the KIR (KEAP1-interacting region) motif of p62 interacts specifically with the Nrf2 inhibitor Keap1 (Kelch-like ECH-associated protein 1) and induces Nrf2 activation upon its liberation from Keap1 [49]. Interestingly, Mallory–Denk bodies, hallmarks of liver pathogenesis, contain Ser349 phosphorylated p62 as well as Keap1 [50]. Recently, a murine splice variant of p62 was identified lacking the KIR motif [51]. Although this variant is an active autophagy receptor, it is not able to degrade Keap1, and contrary to full-length p62, inhibits Nrf2.

Acetylation of p62 by TIP60 and deacetylation by HDAC6 in the UBA domain at Lys420 and Lys435 regulate binding of p62 to ubiquitin. This increases p62-dependent selective autophagy as well as the assembly of p62 bodies, cytoplasmic aggregates containing ubiquitinated proteins and p62 [52]. Other publications suggest that p62 forms structures with more dynamic liquid-like properties, termed liquid droplets, which allow an exchange of their components with their environment [13,53]. This phase separation is mediated by the UBA and PB1 domains [1,12].

RIP1-dependent cleavage of p62 by caspase-8 [54] results in a stable variant lacking the carboxy terminal KIR and UBA domains that promotes mTORC1 signaling instead of contributing to autophagy. In addition, caspase-1 cleaves p62 after Asp329 [55].

The cysteine residues Cys105 and Cys113 of human p62 are redox-sensitive. Upon oxidative stress, they form intermolecular disulfide bonds crosslinking p62, and this oligomerization supports autophagy and cell survival [56].

## 3. p62 Is a Cargo Receptor for Autophagy

Autophagy is a central degradative pathway that occurs in all eukaryotic cells and supports survival under stress conditions [57]. At the tissue level, stressors cause inflammation, a complex process that aims to restore tissue homeostasis after its disturbance [58]. Although inflammation is in principle a beneficial and protective response, it can be destructive, when deregulated or/and chronic. Then, inflammation contributes to the pathology of numerous common diseases. Inflammation is also a hallmark in cancer development [59]. Therefore, cellular autophagy is often associated with inflammation but acts in an anti-inflammatory manner [60,61] (see also Section 4.1). Ablation of the essential autophagy-related gene *ATG16L1* in mice causes an inflammatory phenotype characterized by high levels of IL-1β and -18 due to increased inflammasome activation (see Section 4.1) [62,63].

The term autophagy is commonly used for the process of macroautophagy, defined as the formation of a cellular structure surrounded by a double membrane that fuses with lysosomes for the digestion of its content [64]. Autophagy is triggered by adverse conditions, such as starvation, infection, oxidative stress, protein aggregation, or inhibition of mTORC1 (see Section 4.3) [65]. Autophagy is also induced by inhibition of the ubiquitin-proteasome system (UPS), as this causes accumulation of ubiquitinated proteins, whereas inhibition of autophagy dampens the UPS [65]. Both degradative pathways are required for cellular proteostasis and p62 connects both. p62 binds ubiquitinated proteins, such as tau, via its UBA domain and causes their proteasomal degradation [66]. However, together with other proteins, such as NBR1, TAX1BP1 and Optineurin, p62 plays a more important role as a cargo receptor in autophagy [61]. In general, Lys48 ubiquitination targets proteins for proteasomal degradation, whereas those with Lys63-linked ubiquitin are sequestered by p62 and delivered to autophagosomes [5]. In addition, p62 is required for mitophagy, the autophagic degradation of damaged mitochondria [67], and for xenophagy, the autophagic destruction of invading pathogens [61]. It has been reported that the intracellular bacteria *S. typhimurium*, *S. flexneri*, and *M. tuberculosis* are targeted by p62 for delivery to autophagosomes [68,69,70].

**Figure 2:**
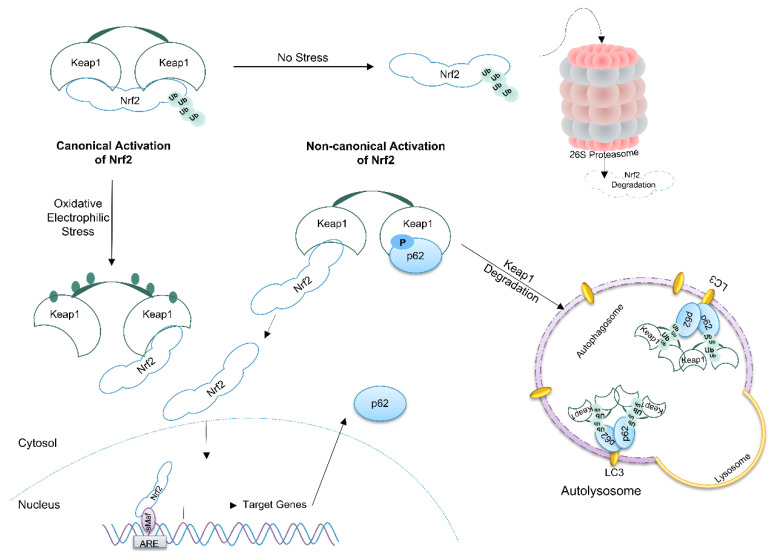
Non-canonical Nrf2 activation by p62. Under non-stressed conditions, the cytoprotective transcription factor Nrf2 is continuously degraded by the proteasome upon Keap1-mediated ubiquitination. Oxidation of regulatory cysteine residues of Keap1 in situations with oxidative and electrophilic stress inhibits Keap1 and ubiquitination of Nrf2 causing canonical Nrf2 activation and induction of target gene expression. When p62 is phosphorylated at Ser349, it binds to Keap1 causing its autophagic degradation. Afterwards, Nrf2 is free to translocate to the nucleus and to induce target gene expression (non-canonical Nrf2 activation) [79].

Under non-stressed conditions, p62 exists in the cell as an inactive homodimer masking its UBA domain from binding to ubiquitin [1,71]. Proteotoxic stress induces phosphorylation of Ser407 by ULK1, causing the liberation of the UBA domain for substrate binding [72]. A subsequent phosphorylation at Ser403 by ULK1, CK2 (casein kinase 2), or TBK1 (TANK-binding kinase 1) enhances the affinity of the UBA domain for ubiquitin [73,74,75]. Upon binding of ubiquitin-conjugated substrates the complex can undergo phase separation by forming a dynamic liquid-like structure [13]. The LIR domain of p62 interacts with LC3 present on autophagosomes (Figure 2). Fusion with lysosomes results in the formation of autolysosomes and the degradation of their content, including p62. Therefore, under conditions where autophagy is impaired, p62 accumulates, and its accumulation levels are proportional to the autophagic impairment, as long as p62 transcription is not strongly downregulated [12,76,77,78].

## 4. Roles of p62 in Different Signaling Pathways

### 4.1. p62 Restricts Inflammasome Signaling

Activation of inflammasomes represents one of the main pathways underlying the induction of an inflammatory response [80]. Moreover, acute and chronic inflammasome activation contributes to the pathogenesis of common diseases and recent research focuses on mechanisms regulating inflammasome activation [81,82]. Increasing evidence demonstrates an important role of p62, autophagy, and the Nrf2/Keap1 pathway (see Section 4.2) in the restriction of the inflammasome pathway [83,84].

**Figure 3:**
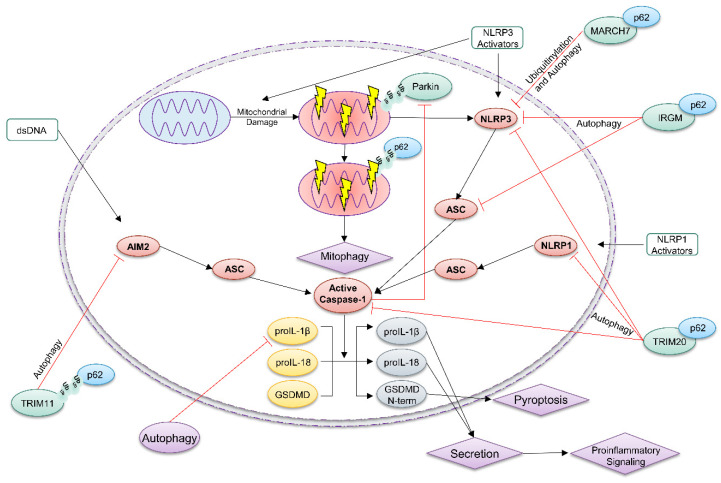
p62-dependent autophagy antagonizes inflammasomes. The inflammasome sensors NLRP3, NLRP1 and AIM2 sense certain stressors, which induce their activation. This causes oligomerization of the adaptor protein ASC (speck formation) and in turn activation of the protease caspase-1 [81]. Subsequently, caspase-1 activates the proinflammatory cytokine proIL-1β and -18 by proteolytic processing. Release of mature IL-1β and -18 induces an inflammatory response, which is dependent on caspase-1-dependent cleavage and activation of GSDMD. p62 is required for mitophagy of damaged mitochondria that induce NLRP3 activation [90]. Moreover, MARCH7 ubiquitinates NLRP3, inducing its p62-dependent selective autophagic degradation [91]. Together with p62, IRGM regulates degradation of NLRP3 and ASC via autophagy [92]. The E3 ubiquitin ligase TRIM20 ubiquitinates NLRP3, NLRP1 and caspase-1, thereby initiating their p62-dependent autophagic clearance [93]. Ubiquitinated TRIM11 binds with p62 to AIM2 causing its degradation by autophagy [94].

Inflammasomes constitute a family of multi-protein complexes that assemble upon sensing of a wide range of stimuli, including DAMPs (damage- or danger-associated molecular patterns) and PAMPs (pathogen-associated molecular patterns) [85]. The sensor protein belongs to the NLR (NOD (nucleotide oligomerization domain)-like receptor) or the ALR (AIM2 (absent in melanoma 2)-like receptor) family. NLRP3 (NLR family pyrin domain containing 3) represents the most prominent inflammasome sensor and its activation is associated with numerous inflammatory diseases, including Alzheimer’s disease, atherosclerosis, and rheumatoid arthritis [86,87]. Once an inflammasome sensor is stimulated by its activator, it recruits the adaptor protein ASC (apoptosis-associated speck-like protein containing a caspase recruitment domain) and induces formation of ASC polymers, termed ASC specks (Figure 3). This causes activation of the protease caspase-1. Subsequently, caspase-1 cleaves and activates the pro-inflammatory cytokines proIL-1β and -18 as well as GSDMD (gasdermin D). Upon oligomerization of its amino terminal fragment, GSDMD forms pores in the outer membrane inducing a lytic type of cell death, termed pyroptosis [88]. As IL-1β and -18 lack a signal peptide for secretion, GSDMD pores play a crucial role in their release and in the induction of an inflammatory response. Whereas the AIM2 inflammasome is activated upon the binding of double stranded pathogen-derived DNA [89], the exact molecular mechanisms underlying NLRP3 activation remain elusive and different models, including pore formation, lysosomal rupture, and mitochondrial dysfunction, are discussed [87].

Upon its role as a cargo receptor in autophagy, p62 negatively regulates the inflammasome pathway by different mechanisms [84,95]. Certain NLRP3 inflammasome activators can damage mitochondria [87], which are then ubiquitinated by the E3 ubiquitin ligase Parkin [96,97]. This recruits p62 targeting them for destruction by the autophagic machinery [90]. Therefore, ablation of p62 expression restricts mitophagy and enhances NLRP3 inflammasome activation [90]. On the other hand, caspase-1 (and caspase-8) antagonizes mitophagy by proteolytic cleavage and inactivation of Parkin [98,99]. Furthermore, p62 ablation disturbs proteostasis, which supports inflammasome activation in primary murine macrophages and in mice in vivo [100].

p62 also directly supports the autophagy-dependent degradation of ubiquitinated inflammasome proteins, such as NLRP3, ASC, and AIM2 (Figure 3) [101]. The E3 ubiquitin ligase TRIM20 targets NLRP3, NLRP1, and caspase-1 for autophagic degradation [93]. MARCH7 ubiquitinates NLRP3, marking it for destruction by autophagy [91]. In contrast, TRIM31 induces NLRP3 degradation through the proteasome [102]. Ubiquitinated TRIM11 binds to AIM2 and recruits p62, thus targeting the inflammasome sensor for destruction by autophagosomes [94]. IRGM (immunity-related GTPase M) supports autophagic degradation of NLRP3 and ASC in a p62-dependent manner and protects from gut inflammation in a mouse model of Crohn’s disease [92]. Recently, Zhou et al. demonstrated that Lys63-linked ubiquitination of NLRP3 drives autophagic degradation of the NLRP3 inflammasome in a p62-dependent manner [103].

The cellular level of proIL-1β, the central inducer of inflammation after inflammasome activation, is regulated by autophagy and it is tempting to speculate that proIL-1β is also targeted by p62 [104]. In addition, polyubiquitinated proIL-1α and -1β are also degraded by the proteasome pathway [105]. As mentioned above, IL-1β lacks a signal peptide and is secreted dependent on GSDMD activation but also by mechanisms which are only partially understood [106]. ProIL-1β binds to TRIM16 [107] and some evidence suggests that, upon this interaction, IL-1β is released from the cell by a mechanism involving autophagy, termed secretory autophagy [108,109].

### 4.2. p62 Activates the Nrf2 Pathway

The transcription factor Nrf2 (nuclear factor E2-related factor 2) is a central regulator of cytoprotection and stress resistance, particularly against ROS and oxidative stress [110,111,112]. Therefore, Nrf2 expression is associated with inflammation and involved in many inflammatory diseases including cancer [113,114]. In general, Nrf2 activation is considered to be anti-inflammatory [115]. p62 activates Nrf2 by a well characterized non-canonical pathway mediated by interaction with and autophagosomal degradation of the Nrf2 inhibitor Keap1 (Kelch-like ECH-associated protein 1) (Figure 2) [79,116]. Furthermore, the Nrf2 pathway is linked to inflammasomes at different levels [117,118].

Nrf2 is a member of the Cap’n’collar family of bZIP (basic leucine zipper) transcription factors and regulates expression of several hundred genes mainly in a positive manner [110,111]. After interaction with sMAFs (small masculoaponeurotic fibrosarcomas), Nrf2 binds to AREs (antioxidant response elements) in the promoter of genes encoding proteins of the glutathione and thioredoxin system, the two most important cellular redox buffers [119]. Furthermore, Nrf2 induces expression of proteins required for detoxification of ROS and xenobiotics. Nrf2 activity is often high in cancer cells, where the transcription factor regulates metabolic reprogramming [23,120]. In addition to these indirect anti-inflammatory effects, Nrf2 is able to repress expression of proinflammatory cytokines, such as proIL1α, -1β, and -6, by a poorly understood mechanism [121]. Transcription of the *Nrf2* gene (*NFE2L2*) is induced by NF-κB, AhR (aryl hydrocarbon receptor), oncogenic pathways, and by Nrf2 itself through a positive feedback loop [112].

The Nrf2 binding partner and inhibitor Keap1 regulates activity of Nrf2 at the posttranslational level. In the cytoplasm, two Keap1 molecules interact with Nrf2 in an asymmetric manner. As Keap1 binds also the E3 ubiquitin ligase complex Cul3/Rbx1 (Cullin 3/RING-box protein 1), this results in constant ubiquitination of Nrf2 and subsequent proteasomal degradation. Consequently, the half-life of Nrf2 is short (about 20 min) and its basic expression level usually low [122]. Keap1 has 27 cysteine residues and some of them are redox sensitive [123]. Oxidation of these cysteines by electrophiles, such as sulforaphane or curcumin, substances found in vegetables, or by glutathione under high ROS conditions, causes a conformational change of Keap1 and an inhibition of Cul3′s E3 ubiquitin ligase activity [111]. After that, newly synthesized Nrf2 bypasses Keap1, translocates to the nucleus, and induces expression of target genes, termed canonical Nrf2 activation (Figure 2).

Binding of p62 via its KIR motif to Keap1 liberates Nrf2 and causes non-canonical Nrf2 activation [79]. The affinity of p62 to Keap1 is strongly increased upon phosphorylation of Ser349 (see Section 3), which occurs while p62 is interacting with cargos [1]. Subsequently, the Keap1-p62-cargo complex is degraded via autophagy (Figure 2). Indeed, Keap1 is degraded mainly by autophagy in a p62-dependent manner [124,125]. Upon p62-induced activation, Nrf2 induces not only the expression of cytoprotective genes, but also the transcription of *SQSTM1*, resulting in a positive feedback loop. However, the Keap1-Cul3-Rbx1 complex can ubiquitinate p62 at Lys420, which causes the degradation of p62 by autophagy [126].

In general, inhibition of autophagy results in accumulation of p62, which in turn activates Nrf2. This pathway plays an important role in the development of liver cancer (see Section 5.). However, Nrf2 levels are high in different types of cancer induced by several mechanisms, including mutations in the *NFE2L2* and *KEAP1* genes or Keap1 inhibition by oncometabolites [110,127]. Cancer cells profit from high Nrf2 activity through increased stress and ROS resistance, but also by metabolic reprogramming [23,120]. On the other hand, high levels of Nrf2 can suppress cancer development and neurodegenerative diseases, demonstrating a dual role of Nrf2 in these conditions [5,127].

### 4.3. p62 Activates mTORC1

Mammalian target of rapamycin (mTOR) is a protein of 289 kDa and a highly conserved member of the PI3K (phosphoinositide 3-kinase)-related kinase family [128]. The serine-threonine kinase plays a complex role as a sensor for the environmental and intracellular status of the cell and regulates central processes, including proliferation, growth, metabolism, and survival [129]. Therefore, the mTOR pathway is an attractive target for immunosuppressive and anti-proliferative therapies for patients after organ transplantation or suffering from cancer, respectively [130,131]. Inhibitors of mTOR, such as rapamycin and rapalogs, are not only tested for their therapeutic potential for cancer patients, but also used in research laboratories as activators of autophagy.

mTORC1 (mTOR complex 1) is composed of the three core proteins mTOR, mLST8 (mammalian lethal with SEC13 protein 8) and Raptor (regulatory-associated protein of mTOR), and the inhibitors PRAS40 (proline-rich AKT1 substrate 1) and DEPTOR (DEP domain-containing mTOR-interacting protein) [130]. In the presence of amino acids, nutrients, energy, and growth factors, mTORC1 induces anabolic processes, such as lipid, nucleotide, and protein synthesis, by phosphorylation of several substrates, thereby supporting cell growth. In addition, mTOR phosphorylates ULK1, a kinase required for initiation of autophagy, leading to the inhibition of catabolic processes [132]. Although p62 acts as a cargo receptor for autophagic degradation, it activates the mTORC1 pathway [133]. p62 interacts with Raptor which is required for assembly, subcellular localization, stability, and substrate recruitment of mTORC1 [130,133]. In the presence of amino acids, MEKK3 (mitogen-activated protein kinase kinase kinase 3) binds to the PB1 domain of p62 and induces phosphorylation of Thr269 and Ser272 [134]. Then, p62 moves to the lysosomal membrane, binds the E3 ubiquitin ligase TRAF6 as well as mTOR, and induces Lys63-linked ubiquitination of mTOR. This p62-TRAF6-dependent ubiquitination causes activation of mTORC1 inducing anabolism and cell growth [134,135].

mTOR is a regulator of immune cell function and contributes to the high proliferation rate of cancer cells [129,131]. Therefore, *MTOR* itself as well as genes coding for upstream activators of mTORC1, such as *AKT1*, *EGFR,* or *PTEN,* are frequently mutated in cancer [130].

### 4.4. p62 Activates NF-κB

The NF-κB (nuclear factor-κB) family of transcription factors consist of five members that act as homo- or heterodimers [136,137]. They play a central role in inflammatory processes, numerous (inflammatory) diseases and are involved in all stages of tumor development [138]. p62 activates the NF-κB pathway by different mechanisms and thereby supports inflammation and cancer development [1,139].

In the classical pathway, translocation of the Rel-p50 dimer is regulated by the IKK (IkappaB kinase) complex, which is composed of the regulatory subunit NEMO (NF-kappa-B essential modulator or IKKγ) and the catalytic subunits IKKα and IKKβ. After activation, the IKK complex phosphorylates IκBα that dissociates from Rel-p50 dimers, gets ubiquitinated and degraded by the proteasome. Activation of IKK is induced by activation of several receptors by binding of their ligands, such as TNFα, IL-1 or LPS. The kinase RIP1 induces activation of the IKK complex, and this is supported by interaction with the ZZ domain of p62 (Figure 1) [41]. Activation requires a dimerization of two IKK units, induced by trans-autophosphorylation and Lys63-linked ubiquitination of NEMO. This process is catalyzed by the E3 ubiquitin ligase TRAF6 and supported by p62 via a direct interaction with TRAF6 [140]. A20 is a negative regulator of NF-κB [141]. This ubiquitin-editing protein prevents Lys63 ubiquitination of TRAF6 and promotes Lys48-linked ubiquitination of RIP1, causing proteasomal degradation of the latter [1,142]. p62 promotes degradation of A20 by autophagy.

NF-κB is also a positive transcriptional regulator of p62 expression and supports its own activity via a feed-forward loop. In addition, the transcription factor plays a central role in priming for inflammasome activation by inducing expression of proIL-1β and NLRP3 (Section 4.1) [143]. Nevertheless, via positive regulation of Nrf2 expression (Section 4.2), NF-κB restricts proIL-1β expression. In addition, the NLRP3 inflammasome pathway is also antagonized by NF-κB upon p62-dependent mitophagy [90,144].

## 5. p62 in Hepatocellular Carcinoma and Other Malignancies

A key role for autophagy, p62, and Nrf2 is well established in the development of different types of cancer, particularly of HCC [145,146]. HCC is the sixth most common cancer and the third leading cause of tumor death.

Different types of stress to the liver and hepatocytes cause liver cirrhosis, a disease characterized by impaired liver function due to the formation of scar tissue as a consequence of chronic tissue repair. This is caused by infection with HBV/HCV (hepatitis B and C virus), chronic alcohol abuse leading to ASH (alcoholic steatohepatitis), NASH (non-alcoholic steatohepatitis) due to obesity, high blood pressure, type 2 diabetes, metabolic syndrome, exposure to chemical carcinogens, or genetic predisposition and diseases [147]. Particularly HBV, HCV, ASH and NASH are known to inhibit autophagy, causing formation of inclusion bodies and accumulation of damaged mitochondria [148]. Low levels of beclin 1 result in autophagy defects in all stages of HCC [149,150]. Beclin 1 is monoallelically deleted and decreased in different cancers, such as prostate, breast and ovarian cancer [151]. Upon decreased rates of autophagy, phosphorylated p62 accumulates with Keap1 in structures of the human liver, indicating Nrf2 activation that contributes to adenoma formation and the development of HCC [152,153]. This is associated with increased NF-κB, mTOR and Wnt/β-catenin signaling. Therefore, p62 represents a hub linking defects in autophagy with critical pathway regulating carcinogenesis, such as inflammation, redox homeostasis and (energy) metabolism [145]. Particularly, metabolic reprogramming supports tumor growth as well as its drug resistance [23,154]. The expression of key players in autophagy provides useful indicators for the prognosis of patients suffering from HCC. High levels of LC3B and ULK1 are associated with a poor prognosis of HCC patients in advanced stages [155,156]. Sorafenib, a tyrosine kinase inhibitor, is a standard drug for liver cancer that enhances autophagy [157,158]. Furthermore, mTOR inhibition by rapamycin/sirolimus has promising anti-tumoral effects in HCC patients [159].

Experiments in mice have confirmed the central role of the autophagy-p62-Nrf2 axis in the development of HCC [1]. Loss of Atg5 (autophagy related 5) or Atg7 in the mouse liver is associated with damaged mitochondria and peroxisomes, lipid droplets and accumulation of protein aggregates with p62 and Keap1, leading to sustained Nrf2 activation that causes benign adenoma [160,161]. Similarly, mice haploinsufficient for beclin 1 are prone to spontaneous tumor development. Furthermore, they are highly susceptible to develop HCC upon infection with HBV [162]. Most likely, ROS derived from degenerated mitochondria and peroxisomes damage DNA causing genomic instability and promoting oncogenesis [163]. The accumulation of p62 in hepatocytes of these mice is sufficient to induce HCC development and allows enhanced proliferation of cancer cells and resistance to anticancer drugs due to Nrf2 activation [23]. Activation of mTORC1 due to TSC (tuberous sclerosis complex) depletion causes HCC development that is reverted by additional p62 deletion [24]. In addition, the simultaneous loss of p62 or Nrf2 in Atg5- or Atg7-deficient mice suppresses tumor development demonstrating the central role of Nrf2 and p62-induced Nrf2 activation in the development of HCC [160,164]. However, it is also known that NF-κB, which is linked both to p62 and to Nrf2, is activated in human HCC and its inhibition as well as activation in mice enhances hepatocarcinogenesis [165,166,167]. Ablation of p62 in hepatocytes is not associated with defects in autophagy, demonstrating that other autophagy receptors can compensate for the lack of p62 [24]. Nonetheless, p62 expression is essential for mitophagy and suppression of the NLRP3 pathway [90]. As discussed above (Section 3), autophagy is an anti-inflammatory stress-induced pathway. Autophagy plays opposing roles at different stages of tumorigenesis. At early stages, autophagy is rather tumor suppressive and cytoprotective, at late stages, tumor cells might need higher levels of autophagy [145,168,169]. This model is supported by the observation that mice lacking expression of Atg5 or Atg7 in hepatocytes develop benign adenomas but not malignant HCC. Moreover, in rats, inhibition of autophagy promotes hepatocarcinogenesis in the dysplastic stage but suppresses tumorigenesis in the tumor-forming stage of HCC [170].

p62 expression is also upregulated in a number of other tumor cells and this list is most likely further increasing (Table 1). Consequently, pharmacological targeting of p62 might represent a successful anti-tumor strategy. First, bioactive molecules were developed that bind to certain domains of p62 and block the pathways and activities regulated by these domains [171].

## 6. Summary and Outlook

**Figure 4:**
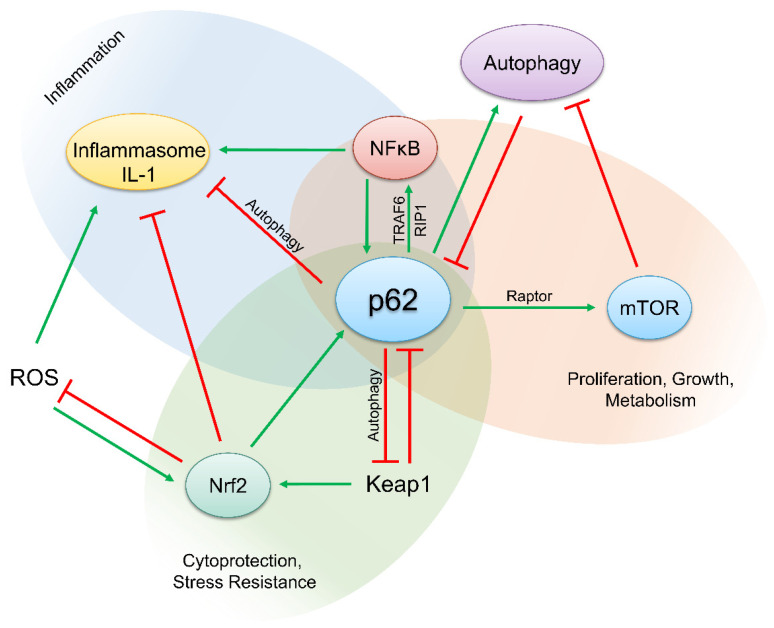
p62 regulates and links central pathways in inflammation and cancer development. p62 is an important cargo receptor for autophagy and mitophagy and thus inhibits inflammasome activation and inflammation. Moreover, upon Keap1 binding, p62 activates Nrf2 and attenuates inflammation. In contrast, p62 activates NF-κB, a central regulator of inflammation and cancer development, and the kinase mTOR that induces anabolic pathways.

The p62 protein is a beautiful paradigm for complexity in molecular biology. Via its domain structure, p62 interacts with several proteins regulating key pathways required for the maintenance of homeostasis of cells and tissues, as well as for inflammation and common diseases including cancer (Figure 4). It is likely that further novel binding partners of p62 that are critically involved in other pathways will be revealed in the future. A recent example is the identification of a role of extracellular p62 as an inflammatory mediator in sepsis [185]. p62 is actively secreted from macrophages and monocytes by secretory lysosomes or passively released by pyroptotic cells and binds to the insulin receptor. This causes NF-κB activation and in turn polarization of macrophages contributing to septic death [186]. There is no need to say that p62 requires a specific posttranslational modification (in form of phosphorylation at Ser403) for this role in sepsis [186]. Although research in the past has revealed that p62 is modified and regulated by phosphorylation, ubiquitination, acetylation, proteolytic processing, and the formation of disulfide bridges, we are far away from precisely understanding how the binding of p62 to its numerous interaction partners is regulated. Furthermore, the physical and biological properties of protein aggregates with p62 are incompletely understood. It is clear that p62 plays an important role in inflammation, several neurodegenerative diseases, and cancer. Particularly, the role of p62 in cancer development seems to be complex and double-faced. This is caused by the different pathways linked to p62. p62 is a cargo receptor in auto-, mito-, and xenophagy, as well as an important hub for pro- and anti-inflammatory pathways, such as inflammasomes, NF-κB, Nrf2, and mTORC1. As these pathways have dual or even multiple and in part opposing roles in cancer development depending on the tissue, cell type, and stage of development, it is difficult to estimate whether or not p62 represents a screw, the turning of which might have therapeutic potential. Nevertheless, vaccines for targeting p62 have been developed and tested in humans [187,188]. In addition, the expression, modification, and localization of p62, perhaps in connection with pathways regulated by p62, might be useful as a diagnostic and prognostic marker in cancer and other diseases [145].

## Figures and Tables

**Table 1:** Human cancers with overexpression of p62 [4].

Cancer Type	References
Thyroid cancer	[172]
Lung cancer	[173]
Colorectal cancer	[174]
Head and neck cancer	[175]
Gastric cancer	[176]
Liver cancer	[24]
Pancreatic cancer	[176]
Renal cancer	[177]
Urothelial cancer	[178]
Prostate cancer	[179]
Breast cancer	[180]
Ovarian cancer	[181]
Endometrial cancer	[182]
Melanoma	[183]
Glioma	[184]
